# The predictive role of Ki67 in pathological complete response (pCR) and invasive disease-free survival (IDFS) in HER2-positive breast cancer: a bi-centric retrospective cohort study of 244 cases

**DOI:** 10.1007/s00404-026-08401-7

**Published:** 2026-05-02

**Authors:** Laura Weydandt, Saida Agabejli, Massimiliano Lia, Pauline Wimberger, Bahriye Aktas, Theresa Link

**Affiliations:** 1 Department of Gynecology, University Hospital Leipzig, Leipzig, Germany; 2 Partner Site Leipzig, Comprehensive Cancer Center Central Germany, Leipzig, Germany; 3 National Center for Tumor Diseases (NCT), NCT/UCC Dresden, a partnership between DKFZ, Faculty of Medicine and University Hospital Carl Gustav Carus, TUD Dresden University of Technology, and Helmholtz-Zentrum Dresden-Rossendorf (HZDR), Dresden, Germany; 4 Department of Gynecology and Obstetrics, Medical Faculty and University Hospital Carl Gustav Carus, Technische Universität Dresden, Dresden, Germany

**Keywords:** Early breast cancer, HER2, pCR, Ki67

## Abstract

**Purpose:**

The purpose of this study was to examine whether Ki67-scores have a predictive significance for pathological complete response (pCR) and invasive disease-free survival (IDFS) in HER2-positive breast cancer.

**Methods:**

This retrospective, bi-centric cohort study focused on HER2-positive early breast cancer patients undergoing neoadjuvant chemotherapy from 2015 to 2023. Multivariable logistic regression was used to find independent association between various clinical parameters, including Ki67, and pCR. Ki67-values were categorized into three groups (low ≤ 15%, intermediate 15–35%, high > 35%). Kaplan–Meier estimator calculated differences in IDFS.

**Results:**

The study included 244 patients with known Ki67-expression. 147 patients (60.3%) achieved pCR. When categorized, 18 (7.4%) were Ki67 low, 114 (46.7%) Ki67 intermediate and 112 (45.9%) Ki67 high. No correlation between Ki67-score as continuous variable and pCR was observed (p = 0.25). HER2 immunohistochemistry (IHC) score 3 + significantly increased pCR compared to IHC score 2 + (63.2% vs. 45%, p = 0.031). Hormone receptor (HR)-positive tumors had a lower pCR rate (53.1% vs. 74.4%, p = 0.001) compared to HR-negative tumors.

5-year IDFS showed no difference between low Ki67 (88.9%; 95% CI 75.5–100%), intermediate Ki67 (82.0%; 95% CI 72.6–92.7%), and high Ki67 (80.9%, 95% CI 70.1–92.3%) subgroups (p = 0.7).

**Conclusion:**

In HER2-positive breast cancer, the Ki67-score showed no association with either pCR or IDFS, thereby questioning its clinical utility. Conversely the HER2 IHC-score and HR-status were predictive indicators for achieving pCR. Clinical decisions in patients with early HER2-positive breast cancer should not be influenced by Ki67-scores, especially not by using cut-offs.

**Supplementary Information:**

The online version contains supplementary material available at 10.1007/s00404-026-08401-7.

## What does this study adds to the clinical work

**Table:** 

Ki67 demonstrated no association with pCR or IDFS in early HER2-positive breast cancer, indicating that it should not guide therapeutic decisions in routine practice. Treatment planning should be informed by more reliable predictive factors.	

## Introduction

Amplification of human epidermal growth factor receptor-2 (HER2), which is associated with both survival and time to relapse, is found in 20–30% of invasive breast cancers [1]. Therefore, biomarkers predicting treatment response and prognosis are of special interest in this subgroup.

Besides grading, the nuclear antigen Ki67 has become the primary parameter to assess tumor proliferation in breast cancer [2] and has been suggested as a prognostic marker [3]. In fact, promising results have been reported when analyzing the association between Ki67 and pCR in patients with HER2-positive breast cancer [2, 4–6]. However, these studies examined limited cohorts (well below 200 cases) and used a wide range of cut-off values for the Ki67-score to assess its significance (i.e. between 17 and 50%) [4, 5, 7–10].

The GeparTrio trial, one of the largest studies in this field, used conventional cut-off values [11] dividing patients with breast cancer into groups with a low (≤ 15%), medium (15–35%), and high (> 35%) Ki67 scoring [12]. For the complete cohort of 1166 breast cancer core biopsies these three Ki67-categories were significantly associated with pCR [13].

Still, the evidence on the clinical value of Ki67-scoring in HER2-positive breast cancer is uncertain. Although Ki67-scores are assessed as continuous values (i.e. 1% to 100%) most researchers divide their study cohorts based on cut-offs, which are either set arbitrarily or chosen based on which value has the best discrimination (i.e. the minimal p-value). However, the latter approach has been shown to yield biased and misleading results [14–16]. Altogether, this leads to a situation where cut-off values for Ki67-scores vary significantly among studies and additionally might have debatable clinical value.

Consequently, the clinical value of the previously proposed Ki67-thresholds needs to be further examined and validated in sufficiently large cohorts of women with HER2-positive breast cancer.

The aim of this study was 1) to examine the prognostic potential of Ki67 on the rate of pCR and 2) to assess the effect of Ki67-score on invasive disease-free survival (IDFS) in early HER2-positive breast cancer and 3) to test the cut-offs for the Ki67-scores (suggested by the investigators of the GeparTrio-trial) [12] in a current study cohort.

## Materials and Methods

### Study design and population

This is a retrospective, bi-centric cohort study of HER2-positive early breast cancer patients diagnosed between January 2015 and December 2023. The databases of the university hospital Dresden and the university hospital Leipzig were searched systematically and all cases with HER2-positive early breast cancer, who received treatment in either of these two centers, were gathered. Clinical, demographic, histopathological, and follow-up data were obtained through a review of patient records. The tumor markers (including Ki67-scores) and receptor status used in this analysis were established by core needle biopsy (CNB) prior to neoadjuvant chemotherapy. Patients with advanced breast cancer and distant metastases were excluded from the analysis.

Ki67 evaluation followed the recommendations of the International Ki67 in Breast Cancer Working Group [17]. The Ki67 index was determined as the percentage of positively stained invasive tumor cell nuclei among the total number of invasive tumor cells assessed. Scoring was performed by experienced breast pathologists using light microscopy in representative areas of invasive tumor tissue. In cases of intratumoral heterogeneity, a global assessment approach was applied, taking areas of higher proliferative activity into account in accordance with international recommendations.

HER2-positivity was defined as immunohistochemically HER2 3 + or HER2 2 + FISH/CISH positive according to current guidelines [18]. A tumor was classified as HR-positive if 1% or more of the tumor cell nuclei showed positive staining for estrogen and/or progesterone receptors [19].

The primary outcomes were the pCR (defined as ypT0/is, pN0) and IDFS. In addition to the effect of Ki67 on the pCR-rate and survival, this study aimed to analyze the possible predictive value for several variables, including HER2 IHC-score, HR-status, grading, tumor size, lymph node metastases and patient’s age. As this study is retrospective in nature and only pseudo-anonymized data were used, no informed consent was obtained. According to national regulations (§ 34 Abs. 1 Sächsisches Krankenhausgesetz, § 15 Berufsordnung), informed consent was not required. The study was reviewed and approved by the ethics committees of Leipzig (416/23-ek) and Dresden (EK-34012024).

### Ki67 quantification

Ki67 quantification is performed using IHC on formalin-fixed, paraffin-embedded (FFPE) tumor tissue sections. The Ki67-score is calculated as the percentage of positively stained tumor cells among the total number of cancer cells within a defined area, using manual counting [20].

### Statistical analysis

Statistical analyses and design of graphics were performed with the software environment R (version 4.4.1., www.r-project.org). Specifically, we used the packages “gtsummary” (descriptive statistics), “rms” (regression analysis), “mice” (sensitivity analysis), “survival” (survival analysis), “survminer” and “ggplot2” (graphs and survival curves).

Ki67-scores were tested as a continuous variable and as a categorical variable by grouping the values along predefined cut-offs as established in the GeparTrio study (low-Ki67 (≤ 15%), intermediate-Ki67 (15–35%) and high-Ki67 (> 35%).

Logistic regression and Cox regression was used to examine the association of Ki67-scores to pCR and IDFS, respectively. Separate models were built for Ki67-score as a continuous variable and Ki67-scores categorized according to the GeparTrio trial [12].

Statistical adjustment for possible confounding was performed by adding the following variables to the multivariable regression models: HER2 IHC score (2 + vs. 3 +), HR-status (negative vs. positive), grading (grade 1 and 2 merged together and compared with grade 3), tumor size (cut-off at 2 cm), lymph node metastases and patient’s age.

Invasive disease-free survival was defined as the time between surgery and a proven disease relapse. Death without evidence of relapse led to censoring of the case at the time of death. Survival was quantified by hazard ratios and compared by means of the log-rank test.

Cases with missing values were excluded from the cohort. To address potential bias due to missing data, we performed a sensitivity analysis. Missing values were imputed using predictive mean matching for continuous variables and logistic/polytomous regression for categorical variables. 20 new datasets were thus generated and each imputed dataset was fitted to the same models as in the primary analysis of the reduced dataset. Estimates and their confidence intervals were combined using Rubin’s rule and compared with those of the primary analysis.

P-values < 0.05 were considered statistically significant.

## Results

### Overview

Between January 2015 and December 2023, a total of 304 patients were treated for early-stage HER2-positive breast cancer in the two centers. After excluding all cases with unknown pCR-status (n = 2) and missing Ki67-levels due to non-assessment (n = 58), 244 patients were included into the final analysis. Of these patients, 147 (60.2%) experienced pCR, 26 (10.7%) patients suffered an event, while median disease-free and overall survival were 37 and 41.5 months, respectively. Patients received different neoadjuvant chemotherapies, either anthracycline-containing or anthracycline-free with dual blockade (trastuzumab / pertuzumab) [21]. In case of non-pCR, patients were treated with post-neoadjuvant therapy with trastuzumab emtansine [22]. HR-positive tumors received adjuvant endocrine therapy for at least five years after completion of chemotherapy [23]. Ki67 values ranged broadly within the cohort, with an interquartile range of 24–50%. Notably, 112 tumors (46%) showed Ki67 values > 35%, and 55 casese (23%) exceeded 50%. Further characteristics of the study population are shown in Table 1.

**Table 1:** Patient characteristics

Characteristic	*N* = 244^1^
Age (years)	51 (40 – 61)
Clinical tumor size	
1	95 (39)
2	119 (49)
3	19 (7.8)
4	11 (4.5)
Clinical lymph node stage*	
0	135 (56)
1	84 (35)
2	16 (6.6)
3	8 (3.3)
Histologic tumor type	
NST	217 (89)
Lobular	11 (4.5)
Ductulolobular	5 (2.0)
Other	11 (4.5)
Clinical tumor size	
< 2 cm	95 (39)
≥ 2 cm	149 (61)
Ki67-score (%)	33 (24 – 50)
Ki67-category	
< 15%	18 (7.4)
15–35%	114 (47)
> 35%	112 (46)
IHC score	
2 +	40 (16)
3 +	204 (84)
Grading	
1	16 (6.6)
2	114 (47)
3	114 (47)
Hormone receptor status	
Negative	82 (34)
Positive	162 (66)
Pathologic complete response	
no pCR	97 (40)
pCR	147 (60)
Invasive disease-free survival (months)	37 (19 – 60)
Center	
Dresden	141 (58)
Leipzig	103 (42)

^1^Median (IQR); *n* (%)

*One patient had unknown clinical lymph node stage

HER2 = Human epidermal growth factor receptor 2, IHC = Immunohistochemistry, pCR = Pathological complete response, NST = No special type.

Pathological complete response.

Univariable analysis showed no significant association between Ki67-scores and the rate of pCR. This was the case for both Ki67-scores as a continuous variable (p = 0.25) and for Ki67 as a categorical variable (p = 0.49 for Ki67 ≤ 15% vs. Ki67 15–35%; p = 0.28 for Ki67 15–35% vs. Ki67 > 35%). This relationship remained non-significant after statistical adjustment for patient’s age, HR-status, HER2-IHC-score, grading, tumor size and lymph node metastases.

Variables with a significant association with pCR in both univariable and multivariable analysis were patient’s age, HER2 IHC-score, HR-status and grading (Table 2).

**Table 2:** Univariable and multivariable logistic regression analyzing the association between Ki67-score (both as continuous and categorical variable) and pathologic complete response. Statistical adjustment was performed for tumor size, grading, hormone receptors, HER2 IHC-score, clinical lymph node involvement, and patient’s age

Variable	Univariable regression		Multivariable regression (Ki67 as continuous variable)		Multivariable regression (Ki67 as categorical variable)	
	OR (95% CI)^1^	p-value	Adjusted OR (95% CI)^1^	p-value	Adjusted OR (95% CI)^1^	p-value
Ki67-score (%)	1.01 (1.0–1.02)	0.25	0.99 (0.98–1.01)	0.42		
Ki67-category						
< 15%	reference				reference	
15–35%	1.43 (0.52–3.92)	0.49			1.25 (0.43–3.62)	0.68
> 35%	1.73 (0.63–4.78)	0.28			1.12 (0.37–3.41)	0.84
Hormone receptor status						
Negative	reference		reference		reference	
Positive	0.39 (0.21–0.69)	0.002	0.49 (0.25–0.94)	0.034	0.51 (0.26–0.97)	0.044
HER2 IHC- score						
2 +	Reference		Reference		Reference	
3 +	2.10 (1.06–4.21)	0.033	2.27 (1.09–4.78)	0.029	2.22 (1.07–4.68)	0.034
Grade						
1–2	Reference		Reference		Reference	
3	2.21 (1.31–3.78)	0.003	2.39 (1.25–4.67)	0.009	2.19 (1.17–4.18)	0.015
Clinical tumor size						
< 2 cm	Reference		Reference		Reference	
≥ 2 cm	0.61 (0.35–1.04)	0.070	0.64 (0.34–1.19)	0.16	0.63 (0.33–1.16)	0.14
Clinical positive lymph nodes						
No	Reference		Reference		Reference	
Yes	0.85 (0.51–1.43)	0.54	0.80 (0.44–1.46)	0.47	0.82 (0.45–1.49)	0.51
Age (years)	0.97 (0.95–0.99)	0.005	0.97 (0.94–0.99)	0.001	0.97 (0.95–0.99)	0.002

^1^*OR* odds ratio, *CI* confidence interval, *HER*2 human epidermal growth factor receptor 2, *IHC* immunohistochemistry

**Fig. 1:**
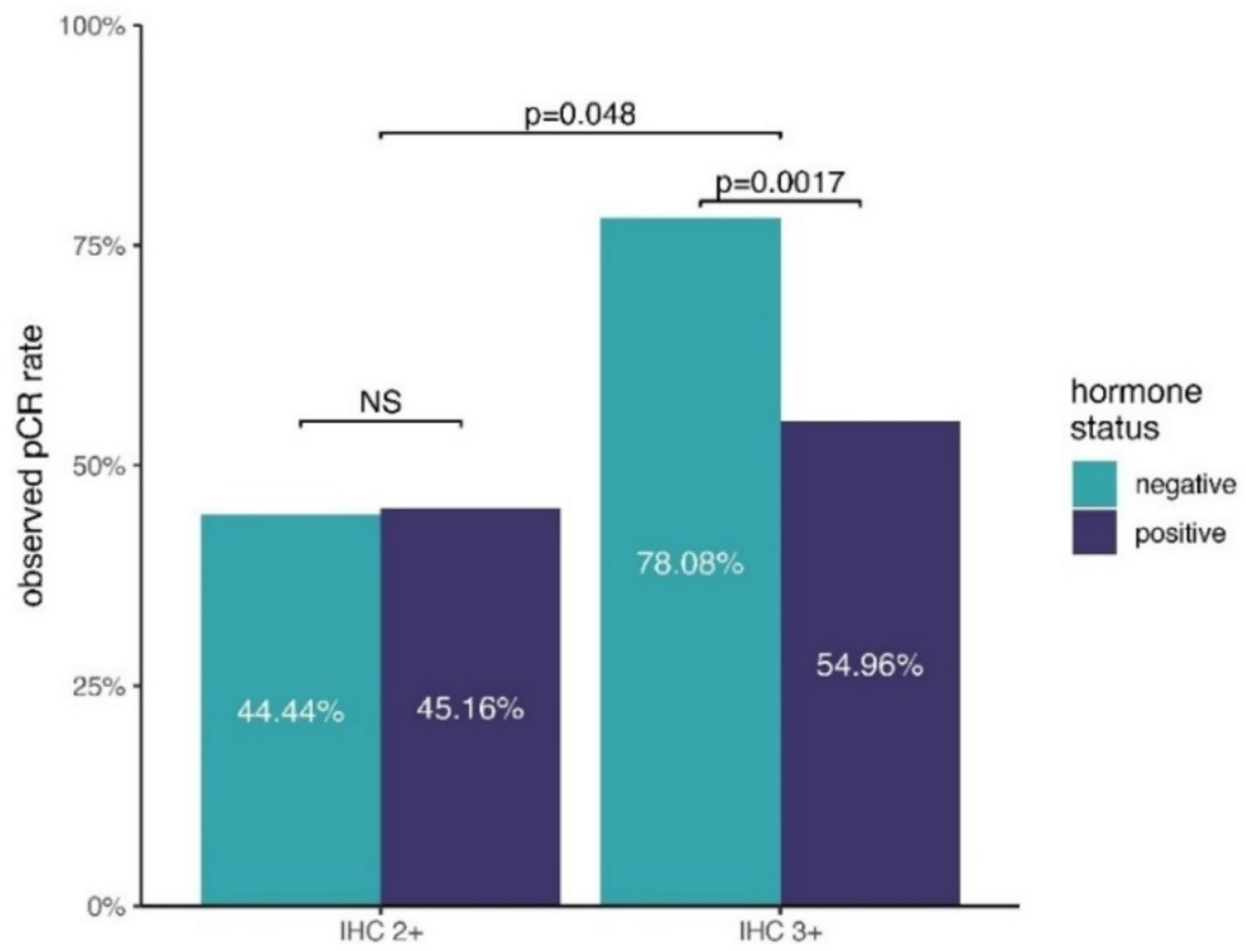
The observed rate of pCR depending on HER2 IHC-score and HR-status. Statistical testing was performed by chi-squared test

A significant association between HER2 IHC-score and pCR was observed (p = 0.048). Furthermore, there was a statistically significant (p = 0.0017) difference in the pCR-rate between HR-positive and HR-negative tumors within the subgroup with a HER2 IHC-score of 3 + . Conversely, such a significant association (p = 1.0) could not be observed in the subgroup with a HER2 IHC-score of 2 + (Fig. 1). The sensitivity analysis showed that these results did not differ relevantly from those yielded by regressions done on data sets with the imputed values. Specifically, there was no change in statistical significance among variables and confidence intervals overlapped (supplementary material).

HER2 = Human epidermal growth factor receptor 2, IHC = Immunohistochemistry, pCR = Pathological complete response, NS = Not significant, HR = hormone receptor.

Survival analysis.

**Fig. 2:**
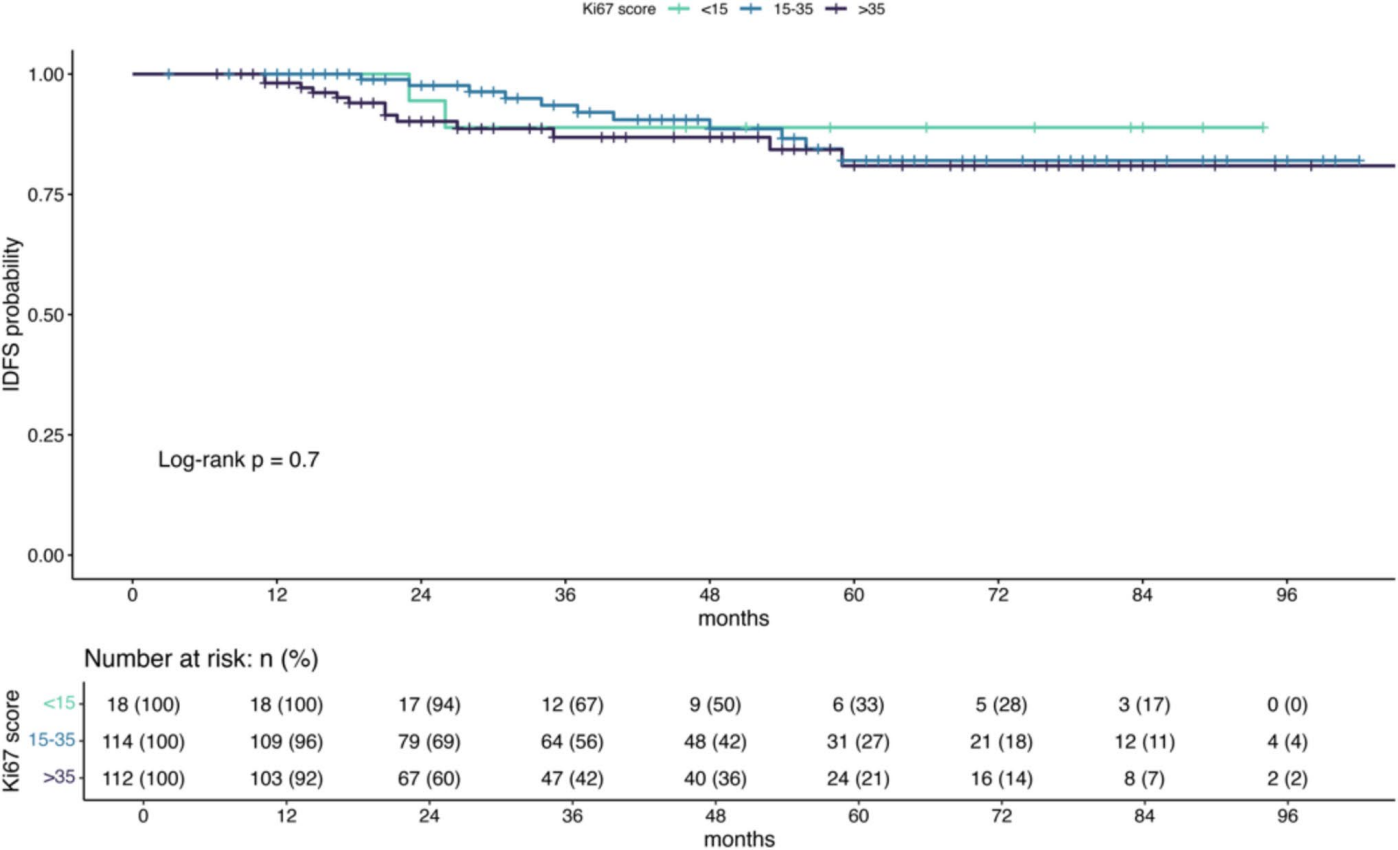
Kaplan–Meier curves of the invasive disease-free survival according to the three different Ki67-categories

Ki67 was not significantly associated with invasive disease-free survival in Cox proportional hazard models. In univariable modeling, this applied to both Ki67 as a continuous variable (p = 0.65) and Ki67 as a categorical variable (p = 0.94 for Ki67 ≤ 15% vs. Ki67 15–35%; p = 0.59 for Ki67 15–35% vs. Ki67 > 35%). Additionally, Ki67 showed no significant association with invasive disease-free survival, either in the subgroup with pCR or in the subgroup without pCR (Table 3). The Kaplan–Meier curve for invasive disease-free survival for the different Ki67 categories is shown in Fig. 2. In the multivariable Cox regression model adjusting for tumor size, grading, hormone receptor status, HER2 IHC-score, clinical lymph node involvement, and age, none of the analyzed variables were significantly associated with invasive disease-free survival (Table 4).

**Table 3:** Ki67 association to invasive disease-free-survival in the two subgroups with pCR and without pCR

	Invasive disease-free survival (Ki67 continuous variable in whole cohort)		Invasive disease-free survival (Ki67 categorical variable in whole cohort)		Invasive disease-free survival (Ki67 continuous variable in cases with pCR)		Invasive disease-free survival (Ki67 categorical variable in cases with pCR)		Invasive disease-free survival (Ki67 continuous variable in cases without pCR)		Invasive disease-free survival (Ki67 categorical variable in cases without pCR)	
Variable	HR (95% CI)^1^	p-value	HR (95% CI)^1^	p-value	HR (95% CI)^1^	p-value	HR (95% CI)^1^	p-value	HR (95% CI)^1^	p-value	HR (95% CI)^1^	p-value
Ki67-score (%)	1.00 (0.99 to 1.02)	0.65			0.99 (0.96 to 1.02)	0.48			1.02 (1.00 to 1.04)	0.12		
Ki67-category												
< 15%			–									
15.1–35%			1.06 (0.23 to 4.77)	0.94								
> 35%			1.50 (0.34 to 6.66)	0.59								
< 15							–				–	
15–35							1.01 (0.12 to 8.38)	> 0.99			1.22 (0.14 to 10.4)	0.86
> 35							0.93 (0.11 to 7.95)	0.95			2.75 (0.34 to 22.1)	0.34

^*1*^*HR* hazard ratio, *CI* confidence interval, *pCR* pathologic complete response

**Table 4:** Multivariable Cox regression models analyzing the association between Ki67-scores (both as continuous and categorical variable) and invasive disease-free survival. Statistical adjustment was performed for tumor size, grading, hormone receptor status, HER2 IHC-score, clinical lymph node involvement, and patient’s age

	Invasive disease-free survival (Ki67 as continuous variable)		Invasive disease-free survival (Ki67 as categorical variable)	
Variable	HR (95% CI)^1^	p-value	HR (95% CI)^1^	p-value
Ki67-score (%)	1.00 (0.98–1.02)	0.82		
Ki67-category				
< 15%			Reference	
15–35%			1.13 (0.24–5.27)	0.88
> 35%			1.30 (0.26–6.38)	0.75
Hormone receptor status				
Negative	Reference		Reference	
Positive	1.13 (0.46–2.81)	0.79	1.20 (0.49–2.96)	0.69
HER2 IHC score				
2 +	Reference		Reference	
3 +	1.31 (0.43–3.98)	0.63	1.28 (0.43–3.88)	0.66
Grading				
1–2	reference		Reference	
3	2.12 (0.87–5.14)	0.10	1.92 (0.80–4.60)	0.14
Clinical tumor size				
< 2 cm	Reference		Reference	
≥ 2 cm	0.58 (0.24–1.38)	0.22	0.57 (0.24–1.36)	0.20
Clinically positive lymph nodes				
No	Reference		Reference	
Yes	2.07 (0.86–4.96)	0.10	2.09 (0.86–5.07)	0.10
Age (years)	0.98 (0.95–1.01)	0.25	0.98 (0.95–1.01)	0.25

^*1*^*HR* hazard ratio, *CI* confidence interval, *IHC* immunohistochemistry

## Discussion

The main finding of the study is that Ki67 did not predict either pathological complete response (pCR) or invasive disease-free survival (IDFS) in patients with early-stage HER2-positive breast cancer. As a secondary finding, we observed a significantly higher rate of pCR in tumors with higher HER2-protein expression.

Several studies have suggested that Ki67 is predictive for pCR in HER2-positive breast cancer. However, most of these studies categorized Ki67-values by establishing and testing their own cut-offs, which varied relevantly across studies (ranging from 17–50%) [4, 5, 7–10]. This variability makes it difficult to compare study outcomes and further calls into question whether any truly “optimal” Ki67-cut-off exists at all. It is important to point out that establishing a reliable Ki67 cut-off remains a challenge and is already much debated in HR-positive/HER2-negative breast cancer, mainly due to the considerable inter- and intra-observer variability in assessment [24, 25]. In addition, standardization remains difficult, as scoring inconsistencies can occur during the pre-analytical, analytical, interpretative, and data analysis phases of Ki67 assessment, making it a complex marker to apply reliably in clinical practice [2].

Furthermore, testing multiple cut-offs and choosing the one with the minimal p-value has been shown to lead to a tenfold increase of false-positive results [14]. As an example, one study reported a threshold between 17–20% where Ki67 was predictive of pCR in patients with HER2-positive breast cancer. However, no statistically significant difference was found when comparing mean Ki67-levels in patients experiencing pCR and those with invasive residual tumor after neoadjuvant chemotherapy [7]. In summary, there are relevant limitations in previous studies, both statistical and methodological, which do not allow a definitive conclusion on whether Ki67-levels have a predictive value for the oncologic outcome in patients with HER2-positive breast cancer.

The GeparTrio trial, which comprises the largest sub-cohort investigating Ki67 in patients with HER2-positive breast cancer, found that pCR (but not IDFS) differed significantly among conventional Ki67-categories [11]. However, also these “conventional” cut-offs are derived from a study, that tested a sequence of thresholds and proposed those with significant discrimination [6]. As discussed before, this approach has inherent statistical problems which lead to biased results [14–16]. Therefore, the cut-off levels proposed by the GeparTrio trial have been called into question [26].

As a secondary finding, we observed a significantly higher rate of pCR in patients with HER2 IHC 3 + tumors compared to those with HER2 IHC 2 + tumors. This effect was independent from other biomarkers (i.e. tumor grade, HR-status and Ki67 scoring) as well as tumor size and patient’s age. These results are in line with those observed in a previous study on patients with HER2-positive breast cancer [10], suggesting that the level of HER2-protein expression detected by IHC seems to be a reliable predictor of response to neoadjuvant chemotherapy. Additionally, we observed an independent association between HR-negative tumors and a higher pCR rate (Table 2). However, this effect seemed to be limited to tumors with IHC 3 + (Fig. 1).

There are several limitations in this study. First, it is a retrospective trial over a period of eight years. Changes in therapy guidelines and personnel occurred during the study period and may have influenced the association between Ki67 levels and the primary outcomes (i.e. pCR and IDFS), although the Ki67-score does not play a role in clinical decision making in HER2-positive breast cancers at our institutions. Second, Ki67-score assessment was performed manually, which has been shown to have limited interobserver agreement [27–29]. Third, the interpretation of Ki67-related findings is constrained by the modest overall sample size. In particular, the Ki67 ≤ 15% subgroup comprised fewer than 20 patients, resulting in a limited reliability of comparisons across Ki67 categories. This distribution likely reflects the generally high proliferative activity characteristic of HER2-positive breast cancer but nonetheless restricts the robustness of subgroup analyses. Additionally, Ki67 assessment was performed by different pathologists across the two centres and within different pathology departments. In contrast to large-scale studies, no central reference pathology was used, which represents a clear limitation.

Nonetheless, the strengths of this study should be emphasized. First, our study cohort is larger than those of most previous studies analyzing Ki67 in HER2-positive breast cancers [30–32]. Additionally, we analyze Ki67-scoring both as continuous and categorical variable without establishing new cut-offs based on our study cohort. In our opinion, this approach is adequate to establish whether Ki67 scoring is associated with oncologic outcomes. Importantly, we test the clinical value of previously proposed cut-off points [6, 13] in order to validate their clinical usefulness in patients with HER2-positive breast cancer.

Since a single, pre-therapeutic Ki67-value does not seem to be predictive for the oncologic outcome in HER2-positive breast cancer, an outlook for future research could involve assessing the concept of dynamic Ki67. This would be – similar to approaches already explored in HR-positive/HER2-negative subtypes – within the framework of ‘window-of-opportunity’ studies [33]. Importantly, this would entail examining whether short-term changes in the Ki67-index during HER2-targeted therapy can predict treatment response. Given the findings of the PHERGain study, where early responders to dual HER2 blockade were potentially spared chemotherapy, such an approach seems promising [34]. In the long term, this could help to establish a de-escalating treatment strategy for selected patients and avoid unnecessary toxicities.

In summary, our results do not support the use of Ki67-scoring in early-stage HER2-positive breast cancer. Previous evidence on cut-off values of this biomarker may be misleading due to methodological issues and the advances in breast cancer therapy. Consequently, clinical decisions in patients with early HER2-positive breast cancer should not be influenced by Ki67-scores, especially not by using cut-offs.

## Supplementary Information

Below is the link to the electronic supplementary material.

Supplementary file1 (DOCX 16 KB)

## Data Availability

The data presented in this study are available upon reasonable request from the corresponding author. The data are not publicly available due to privacy and ethical reasons.
