# Supplementary material for: The predictive role of Ki67 in pathological complete response (pCR) and invasive disease-free survival (IDFS) in HER2-positive breast cancer: a bi-centric retrospective cohort study of 244 cases
Source: Arch Gynecol Obstet. 2026 May 2;313(1):189. doi: 10.1007/s00404-026-08401-7 (PMC13135544; doi:10.1007/s00404-026-08401-7)
Supplement: Supplementary file 1 — Supplementary file1 (DOCX 16 KB) [file 404_2026_8401_MOESM1_ESM.docx]

**Supplementary materials** (sensitivity analysis):

Multivariable regression for pathologic complete response after cases with missing values were excluded (primary analysis, n=244)

| **Characteristic** | **Estimate** | **Adjusted OR** **(95% CI)**^1^ | **p-value** | |
| --- | --- | --- | --- | --- |
| Ki67-score (%) | -0.0065 | 0.99 (0.98 — 1.01) | 0.42 | |
| Hormone receptor status |  |  |  | |
| negative | reference | reference |  | |
| positive | -0.72 | 0.49 (0.25 — 0.94) | 0.034 | |
| HER2 IHC- score |  |  |  | |
| 2+ | reference | reference |  | |
| 3+ | 0.82 | 2.27 (1.09 — 4.78) | 0.029 | |
| Grade |  |  |  | |
| 1–2 | reference | reference |  | |
| 3 | 0.87 | 2.39 (1.25 — 4.67) | 0.009 | |
| Clinical tumor size |  |  |  | |
| < 2cm | reference | reference |  | |
| ≥ 2cm | -0.45 | 0.64 (0.34 — 1.19) | 0.16 | |
| Clinical positive lymph nodes |  |  |  | |
| no | reference | reference |  | |
| yes | -0.22 | 0.80 (0.44 — 1.46) | 0.47 | |
| Age (years) | -0.035 | 0.97 (0.94 — 0.99) | 0.001 | |
| ^1^OR = Odds Ratio, CI = Confidence Interval, HER2 = Human epidermal growth factor receptor 2, IHC = Immunohistochemistry | | | |  |

Multivarialbe regression for pathologic complete response after imputation of missing values (20 datasets with each n=304)

| **Characteristic** | **Estimate** | **Adjusted OR** **(95% CI)**^1^ | **p-value** | |
| --- | --- | --- | --- | --- |
| Ki67-score (%) | -0.0049 | 1.0 (0.98 — 1.01) | 0.49 | |
| Hormone receptor status |  |  |  | |
| negative | reference | reference |  | |
| positive | -0.61 | 0.54 (0.31 — 0.97) | 0.038 | |
| HER2 IHC- score |  |  |  | |
| 2+ | reference | reference |  | |
| 3+ | 0.64 | 1.89 (1.01 — 3.54) | 0.047 | |
| Grade |  |  |  | |
| 1–2 | reference | reference |  | |
| 3 | 0.67 | 1.95 (1.10 — 3.46) | 0.02 | |
| Clinical tumor size |  |  |  | |
| < 2cm | reference | reference |  | |
| ≥ 2cm | -0.15 | 0.86 (0.5 — 1.47) | 0.58 | |
| Clinical positive lymph nodes |  |  |  | |
| no | reference | reference |  | |
| yes | -0.29 | 0.74 (0.44 — 1.25) | 0.25 | |
| Age (years) | -0.031 | 0.97 (0.95 — 0.99) | 0.001 | |
| ^1^OR = Odds Ratio, CI = Confidence Interval, HER2 = Human epidermal growth factor receptor 2, IHC = Immunohistochemistry | | | |  |
